# Genetic, Clinical, Epidemiological, and Immunological Profiling of IgG Response Duration after SARS-CoV-2 Infection

**DOI:** 10.3390/ijms25168740

**Published:** 2024-08-10

**Authors:** Flávia Póvoa da Costa, Kevin Matheus Lima de Sarges, Rosilene da Silva, Erika Ferreira dos Santos, Matheus Holanda do Nascimento, Alice Maciel Rodrigues, Marcos Henrique Damasceno Cantanhede, Fabíola Brasil Barbosa Rodrigues, Maria de Nazaré do Socorro de Almeida Viana, Mauro de Meira Leite, Camille Ferreira de Oliveira, Pablo Fabiano Moura das Neves, Gabriel dos Santos Pereira Neto, Mioni Thieli Figueiredo Magalhães de Brito, Andréa Luciana Soares da Silva, Daniele Freitas Henriques, Juarez Antônio Simões Quaresma, Luiz Fábio Magno Falcão, Maria Alice Freitas Queiroz, Izaura Maria Vieira Cayres Vallinoto, Antonio Carlos Rosário Vallinoto, Giselle Maria Rachid Viana, Eduardo José Melo dos Santos

**Affiliations:** 1 Laboratory of Genetics of Complex Diseases, Institute of Biological Sciences, Federal University of Pará, Belém 66075-110, Pará, Brazil; flaviacosta@ufpa.br (F.P.d.C.); kevsarges@gmail.com (K.M.L.d.S.); rosilenesilva2005@yahoo.com.br (R.d.S.); erikaferreira.bio@gmail.com (E.F.d.S.); nmatheus954@gmail.com (M.H.d.N.); alice.maciel@icb.ufpa.br (A.M.R.); ma_cantanhede@hotmail.com (M.H.D.C.); fbrasil.barbosa@yahoo.com.br (F.B.B.R.); maryvyana@hotmail.com (M.d.N.d.S.d.A.V.); mauroleite@ufpa.br (M.d.M.L.); mionibrito@gmail.com (M.T.F.M.d.B.); dealuciana@gmail.com (A.L.S.d.S.); 2 Graduate Program in Biology of Infectious and Parasitic Agents, Federal University of Pará, Belém 66075-110, Pará, Brazil; gabrielnetoenf@gmail.com (G.d.S.P.N.); alicefarma@hotmail.com (M.A.F.Q.); ivallinoto@ufpa.br (I.M.V.C.V.); vallinoto@ufpa.br (A.C.R.V.); giselleviana@iec.gov.br (G.M.R.V.); 3 Section of Arbovirology and Hemorrhagic Fevers, Evandro Chagas Institute, Health Surveillance Secretariat, Brazilian Ministry of Health, Ananindeua 67000-000, Pará, Brazil; camille.oliveeira@gmail.com (C.F.d.O.); danielehenriques@iec.gov.br (D.F.H.); 4 Center of Biological and Health Sciences, State University of Pará, Belém 66050-540, Pará, Brazil; pablo@uepa.br (P.F.M.d.N.); juarez.quaresma@gmail.com (J.A.S.Q.); fabiofalcao@uepa.br (L.F.M.F.); 5 Laboratory of Virology, Institute of Biological Sciences, Federal University of Pará, Belém 66075-110, Pará, Brazil; 6 Malaria Basic Research Laboratory, Parasitology Section, Evandro Chagas Institute, Health Surveillance Secretariat, Brazilian Ministry of Health, Ananindeua 67000-000, Pará, Brazil

**Keywords:** COVID-19, immunology, antibodies, host genetics

## Abstract

The IgG response against SARS-CoV-2 infection can persist for over six months (long response; LR). However, among 30% of those infected, the duration can be as short as three months or less (short response; SR). The present study assembled serological data on the anti-SARS-CoV-2 IgG response duration of two previous studies and integrated these results with the plasmatic cytokine levels and genetic profile of 10 immune-relevant SNPs that were also previously published, along with the plasmatic total IgG, IgA, and IgM levels, allowing for the genetic, clinical, immunological, and epidemiological aspects of the post-COVID-19 IgG response duration to be understood. The SR was associated with previous mild acute COVID-19 and with an SNP (rs2228145) in *IL6R* related to low gene expression. Additionally, among the SR subgroup, no statistically significant Spearman correlations were observed between the plasma levels of IL-17A and the Th17 regulatory cytokines IFN-γ (rs = 0.2399; *p* = 0.1043), IL-4 (rs = 0.0273; *p* = 0.8554), and IL-2 (rs = 0.2204; *p* = 0.1365), while among the LR subgroup, weaker but statistically significant Spearman correlations were observed between the plasma levels of IL-17A and IFN-γ (rs = 0.3873; *p* = 0.0016), IL-4 (rs = 0.2671; *p* = 0.0328), and IL-2 (rs = 0.3959; *p* = 0.0012). These results suggest that the Th17 response mediated by the IL-6 pathway has a role in the prolonged IgG response to SARS-CoV-2 infection.

## 1. Introduction

SARS-CoV-2 causes a respiratory disease called COVID-19. The COVID-19 disease caused a significant increase in hospitalizations for pneumonia with multiorgan disease, compromising public health and medical services around the world [1]. 

Since the beginning of the COVID-19 pandemic, numerous mutations of SARS-CoV-2 have been identified. Periodic viral genomic sequencing helps to detect new genetic variants circulating in communities. An updated version of the SARS-CoV-2 phylogenetic tree is shared on the GISAID platform (Global Initiative on Sharing Avian Influenza Data).A variant is recognized as a Variant of Concern (VOC) or Variant of Interest (VOI) by the World Health Organization (WHO) [2].

It is well known that the genome of SARS-CoV-2 (~30 kb) encodes 16 non-structural proteins (NSPs) and 4 main structural proteins, including spike (S), envelope (E), core membrane (M), nucleocapsid (N), and other accessory proteins [2].

The SARS-CoV-2 antibodies target the two subunits S1 and S2, RBD, and N. Immunoglobulin G targeting N proteins (anti-N IgG) is detectable in the serum of infected patients, whereas IgG targeting the S1 subunit protein (anti-S1 IgG), S2 subunit protein (anti-S2 IgG), and RBD (anti-RBD IgG) is detectable in the serum of infected or vaccinated patients [2].

The SARS-CoV-2 infection displays a wide spectrum of outcomes, from a deadly disease to asymptomatic conditions, and can also induce heterogeneous patterns of antibody response regarding both the intensity and duration of detectable IgG persistence. Some patients do not develop any detectable IgG after virus infection, while others present long-term IgG persistence over many months [3,4,5].

Despite some evidence suggesting that antibody response decays, in general, 6–8 months after infection or vaccine [4,6,7], previous studies were able to report that anti-Spike and anti-RBD IgG became undetectable or very low before three months after infection in up to 30% of the patients [8,9,10,11,12,13].

The mechanisms and associated factors underlying the heterogeneity of the IgG response duration after SARS-CoV-2 infection are poorly understood, but it is probable that genetic backgrounds have a role, as some studies have associated gene polymorphisms in the IL-6 pathway [14,15], among other pathways, with the COVID-19 IgG response in vaccination [16,17], along with other biological factors like comorbidities and COVID-19 severity [18,19,20]. Hence, the present study assembled serological data on the anti-SARS-CoV-2 IgG response duration of two previous studies and integrated these results with the plasmatic cytokine levels and genetic profiles of 10 immune-relevant SNPs that were also previously published, as well as with the plasmatic total IgG, IgA, and IgM levels, allowing the genetic, clinical, immunological, and epidemiological aspects of the post-COVID-19 IgG response duration to be understood.

## 2. Results

### 2.1. Clinical and Epidemiological Profiling of IgG Response Duration

The short IgG response duration could be associated with the male sex, along with hospitalization and moderate/severe acute COVID-19. Moreover, there was a trend toward the frequency of COVID-19 symptoms to be associated with a long IgG response (Table 1).

Otherwise, age, ethnicity, and comorbidities showed no significant association with the duration of the IgG response (Table 1).

### 2.2. Genetic Factors in IgG Response Duration

In Table 2, the allele and genotypic frequencies of the two groups analyzed are shown, and it is observed that there is an association between the LR group and *IL6* and *IFNG* when compared to the control group. The short response group showed an association with *IL6R* and *IFNG.*

### 2.3. Cytokine Profiles in IgG Response Duration

**Figure 1:**
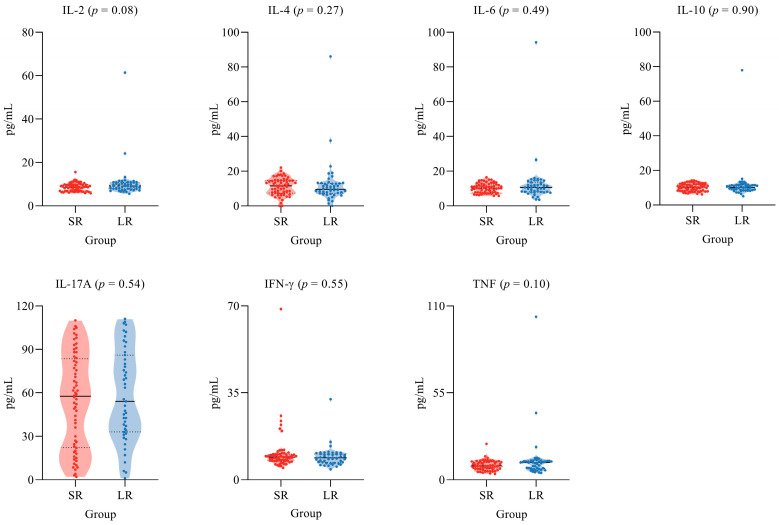
Graphics showing the seven cytokine profiles in the IgG response duration. The black lines represent the median values, and the dashed lines represent the quartiles.

**Figure 2:**
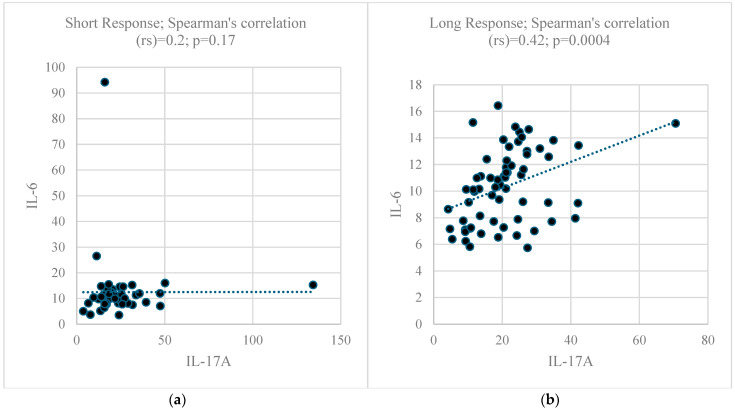
Dispersion plots show (**a**) the absence of correlation between plasma levels of IL-6 and IL-17A in the Short response group; and (**b**) a statistically significant correlation between plasma levels of IL-6 and IL-17A in the Long response group Dots are samples with values in the x and y axis and correlation trends are indicated by dash lines. Additionally, among the SR subgroup, no statistically significant Spearman correlations were observed between the plasma levels of IL-17A and IFN-γ (rs = 0.2399; *p* = 0.1043), IL-4 (rs = 0.0273; *p* = 0.8554), and IL-2 (rs = 0.2204; *p* = 0.1365). Furthermore, among the LR subgroup, weaker but statistically significant Spearman correlations were observed between the plasma levels of IL-17A and IFN-γ (rs = 0.3873; *p* = 0.0016), IL-4 (rs = 0.2671; *p* = 0.0328), and IL-2 (rs = 0.3959; *p* = 0.0012).

The plasmatic levels of seven cytokines are shown in Figure 1, and no statistical differences were observed, as addressed by the Mann–Whitney test. On average, the levels of IL-17A were approximately twice the levels of the remaining cytokines. As IL-6, IFN-γ, IL-4, and IL-2 are the key regulators of IL-17A, a marker of Th17 response, the correlation between IL-17A and the other four cytokines was determined, demonstrating statistical significance for all correlation tests carried out in the LR subgroup, but there was a lack of significance in all correlation tests carried out in the SR subgroup, as presented in Figure 2.

### 2.4. Total Plasmatic IgG, IgA, and IgM Levels

**Figure 3:**
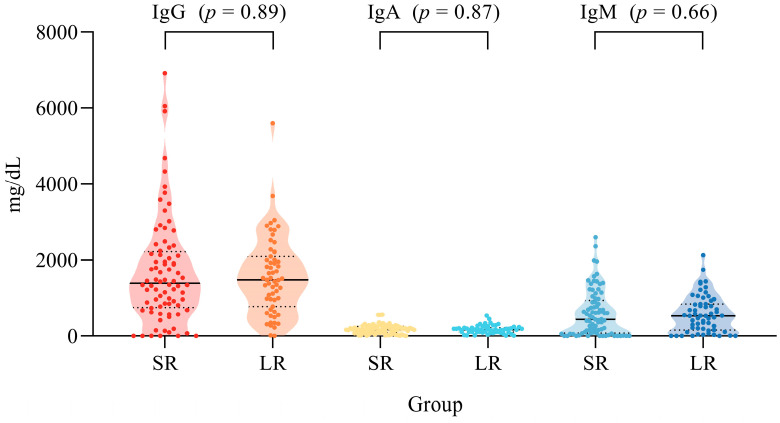
Total plasmatic levels of total IgG, IgA, and IgM among SR and LR subgroups of patients. Black lines represent median values, and dashed lines represent quartiles.

The plasmatic levels of total immunoglobulins (IgG, IgA, and IgM) between both the LR and SR subgroups showed no statistical differences, as addressed by the Mann–Whitney test (Figure 3). Additionally, considering the datasets of Soares et al. [11] and Oliveira et al. [12] separately, there is no correlation between the anti-SARS-Cov-2 IgG levels and total IgG, IgA, and IgM plasmatic levels, as addressed by Spearman’s correlation (Table 3).

## 3. Discussion

Before discussing the results, it is important to provide the historical context in which this study was carried out. At the beginning of the pandemic, a task force was assembled from several fronts, namely research groups at the Federal University of Pará, the Long COVID care service from State University, and research groups at Institute Evandro Chagas, a reference center for research and surveillance in public health. During the pandemic, research was carried out while the disease evolved, generating many epidemiological scenarios, like the first wave in 2020, the many virus strains in 2021 along with the vaccine that had significant coverage in Brazil by the end of 2021, and the advent of Omicron in an admix of vaccinated/non-vaccinated scenarios in 2022. Hence, across these scenarios, the most relevant scientific questions were not the same.

In the beginning, the factors determining the disease severity were the major questions, along with immune profiling, and the immunization behavior by previous disease because of the urgency of vaccine development. These issues were a priority in the first studies published by our group that provided the initial data used in the present study: the studies by Oliveira et al. (submitted for publication in February 2022) and Queiroz et al. (submitted for publication in April 2022) [12,21]. The first study used a cohort of 60 individuals and described the behavior of immunization by SARS-CoV-2 infection, and the second described cytokine profiling. At this time point, these questions were highly relevant, and it is important to highlight that data on the putative factors are not yet available.

Only in early 2023 did additional genetic and serological data come out in the papers by Silva et al. (submitted in March 2023) and Soares et al. (submitted in February 2023). Both studies also had very specific goals, including evaluating IgG profiling in disease severity and long-term COVID-19 development, as well as the genetic influence in long-term COVID-19 development. The completion of these studies allowed for the sample size of IgG profiling (that was only 60 in Oliveira et al.’s study) [12] to be increased and for a genetic data [22] layer to be added, allowing diverse kinds of data (genetic, cytokine, clinical, and epidemiological) to be assembled around a larger well-characterized IgG sample. Only then did addressing the factors influencing these profiles become possible. However, we detected that it would be necessary to evaluate the total immunoglobulin level profiles as well as to gather more genetic data for some loci in a sample before the COVID-19 outbreak. These gaps were filled in 2023.

The results present an association between a short IgG response duration and hospitalization and moderate/severe acute COVID-19 along with the male gender, which has also been associated with disease severity [23,24], suggesting that these associations with gender and disease severity are not independent. There were several studies concerning the duration of antibody response against SARS-CoV-2 infection, aiming to understand how long the IgG response can persist [6,25,26,27]. However, an association between a short or no IgG response and severity was not yet approached in the literature, putatively because they were focusing on investigating the prolonged response in the scenario of immunization by a previous infection or by a vaccine.

An apparent paradoxical result is the enrichment of symptoms among the LR subgroup. We reappraise the data from Bichara et al. [13], also finding the same pattern, with the enrichment of symptoms among LR individuals (Figure S1; Wilcoxon paired test; Z = 3.23; *p* = 0.0012). In previous studies by our group with larger samples from the same population, there was no enrichment of symptoms among hospitalized patients and those with severe COVID-19 (Table S1) [14,28]. There is an association of the IgG response duration with the enrichment of COVID-19 symptoms that seems to be an independent trend, which was also corroborated by Bichara´s data [13].

Indeed, COVID-19 symptoms have been, in a heterogeneous way, associated with disease severity. While some symptoms, like taste and scent impairment, have been associated with milder disease, others, like respiratory ones, have been more linked to severe outcomes [29,30,31]. Thus, the number and frequency of symptoms are not directly related to the severity of the disease.

Regarding the association of the IgG response duration with genetic markers, the AA genotype frequency of *IFNG* was statistically higher among the controls than among both the LR and SR subgroups. This result agrees with a previous study suggesting an association of this genotype with a lack of symptom development in patients with COVID-19 [27]. Additionally, a weak association was found between the CC genotype of *IL6* and LR. The associations with both *IL6* and *IFNG* were weak and did not remain significant after correcting for multiple tests, suggesting that they can be spurious.

Otherwise, the genotype AA of *IL6R* (rs2228145) showed a stronger association with the SR subgroup. This genotype has been associated with lower levels of soluble IL-6 receptor, as well as a slight increment in plasmatic IL-6 in COVID-19 [32,33].

Together, several studies were able to detect associations of IL-6 pathway genes with COVID-19 severity, long COVID development, as well as with antibody response [34,35,36], highlighting IL-6 pathway SNPs as relevant candidate genetic markers in COVID-19 aspects, including in IgG response duration.

IL-6 is a growth factor for B cells and can induce B cell maturation, and survival, regulating IL-21 expression, which stimulates IgG production by B cells [37,38,39,40]. Hence, this polymorphism, by lowering IL-6 levels, may play a role in the premature lack of anti-SARS-CoV-2 IgG response.

Notably, the IL-6 receptor is a key player in developing the Th17 response pattern, as demonstrated by some authors, as the IL-6 signaling pathway, along with TGF-β, induces Th17 cell development via STAT3 [41]. Additional studies also highlight that the increase in IL-6 receptor expression contributes to Th17 response formation in patients with chronic hepatitis B [42]. In this scenario, we could detect an enhancement in the plasmatic levels of IL-17A in both the LR and SR groups, which showed to be about twice the levels of other cytokines (Figure 1).

The remaining SNPs, despite their roles in several aspects of immune response and thrombophilia, did not show any significant association with IgG response duration. However, previous studies were able to detect that they had some significant associations with either COVID-19 severity or long COVID development [14,22,28,43,44].

The role of Th17 in sustained antibody response was demonstrated in several previous studies involving vaccine response, including the COVID-19 vaccine [45,46]. Despite the relevance of Th17 response in vaccines [47,48,49], our results should be carefully considered because (i) they are related to immunization by a previous disease, and (ii) since 2022, the epidemiological scenario has been dominated by Omicron strains, with higher transmissibility and reinfection rates, even among vaccinated individuals [50]. However, the results provide a clue for further investigations of the role of Th17 response, dependent on IL-6, in the immunization dynamics involving SARS-CoV-2 infection.

Although no statistical differences in the cytokine levels were observed between the LR and SR subgroups, the correlation patterns between IL-17A, a key cytokine released by Th17 cells, and cytokines involved in Th17 response regulation, like IL-6, IFN-γ, IL-4, and IL-2, were different. While statistically significant correlations could be observed among the LR patients, no significant correlations were found among the SR patients (Figure 2).

The literature highlighted that there are two pathways in the development of Th17 response, with one being IL-6-dependent and the other being independent of IL-6 [51]. Thus, the different pattern of correlation between IL-17A levels and their regulatory cytokines, including IL-6, might mean that Th17 cells can play an important role in prolonged IgG response and that this Th17 response may be induced in an IL-6-dependent way. This putative interpretation of the cytokine results is also in agreement with the findings that the *IL6R* genotype AA, which is related to a lower expression of this receptor, was associated with SR, and the presence of the allele C (higher expression allele) seems to be associated with the LR.

The association observed between the *IL6R* polymorphism and IgG response duration cannot be attributed to genetic differences between the control and SR groups due to ethnic composition or some kind of sampling stratification because of the similarities between the allele and genotypic frequencies across all subgroups observed in all remaining seven polymorphisms studied in the *TNF* (rs1800629), *MTHFR* C677T, (rs1801133), *FV* Leiden (rs6025), *CD209* (rs4804803), *CIITA* (rs3087456), *HLA-DPA1* (rs3077), and *HLA-DPB1* (rs9277534) genes.

Finally, we could not identify any influence of variation in the constitutive production of total immunoglobulin in plasma in the anti-SARS-CoV-2 IgG response duration, and there is no detectable correlation between the total immunoglobulin levels and viral IgG response.

The present study approaches the understanding of mechanisms underlying the IgG response duration during the first epidemiological scenario of COVID-19 pandemics, composed of pre-Omicron SARS-CoV-2 lineages and without vaccine immunization. Currently, the epidemiological scenario is quite different, with Omicron-derived lineage predominance and large-scale vaccine-induced immunization. This makes it difficult to confirm or refute our results; however, our motivationwas to reevaluate raw data from previous independent studies that did not addressthe issue of response duration and complement them with assays using stored biological materials to provide stronger evidence of the biological mechanisms behind whether the IgG response to SARS-CoV-2 infection is sustained.

## 4. Materials and Methods

### 4.1. Study Design and Data Sources

Previous studies evaluated the dynamics of the IgG response against SARS-CoV-2 and detected a wide spectrum of response duration and intensity [9,10,11]. For two of them, the samples were also genotyped for immunogenetic markers and measured for cytokine levels.

**Figure 4:**
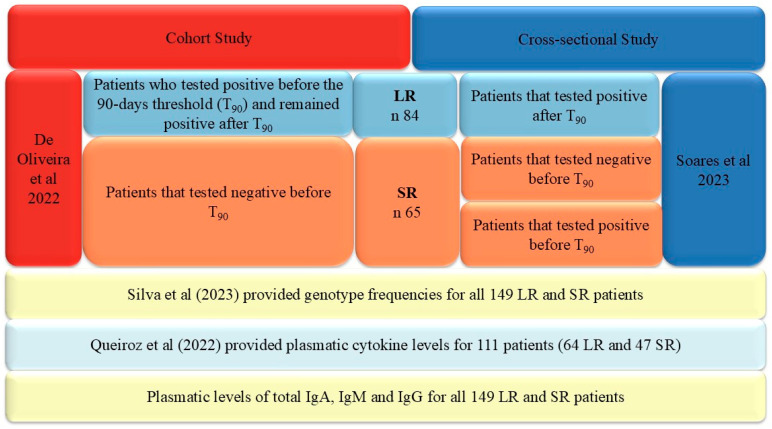
Description of sample design and data sources. LR = Long response subgroup; SR = Short response subgroup. The sources of data were previous studies by De Oliveira et al. [12], Soares et al. [11], Silva et al. [22], and Queiroz et al. [21]. Additionally, in the same samples, we obtained data on the plasmatic levels of the total IgG, IgA, and IgM measured by an ELISA, along with genotypic data of four SNPs (rs2228145/*IL6R*; rs3087456/*CIITA*; rs3077/*HLA-DPA1*; and rs9277534/*HLA-DPB1*) in the Belém representative population that was not reported by Silva et al. [22].

The first was the cohort study by De Oliveira et al. [12] that conducted a follow-up of the IgG response against RDB antigens from the SARS-Cov-2 spike protein using ELISA. Based on this follow-up, they were able to divide the sample of 60 patients into two groups. We used 90 days as a threshold to discriminate short and long IgG response duration based on previous studies that reported a lack of response before three months [52]. As demonstrated in Figure 4, two groups could be identified: (i) 38 patients showed a positive response over 90 days, i.e., detectable IgG, and they were classified as long response (LR), and (ii) 22 patients had a negative response before and after 90 days, i.e., no detectable IgG or a lack of IgG detection before 90 days. They were classified as short response (SR).

The cutoff of three months was chosen because previous studies from our groups, like the one by Bichara [13], used this cutoff. Thus, we adopted the same cutoff to make the data from different studies comparable. Moreover, as we approached the association with long-term COVID-19, the period of 90 days was important, because this time is adopted to classify long COVID (the persistence of symptoms after 90 days or more, without other causes). The use of shorter or longer cutoffs would interfere with the association approach.

The second study by Soares et al. [11] was a cross-sectional study that evaluated the IgG response against the spike protein in different groups of patients, defined by disease severity and the persistence of symptoms. For all patients, the time between COVID-19 and the IgG level measurement by the ELISA was available. Hence, it was possible to identify three groups of patients by using the 90-day threshold: (i) 46 patients had a detectable IgG response after 90 days and were classified as LR, (ii) 7 patients had an undetectable IgG response before 90 days and were classified as SR, and (iii) 36 patients had a detectable IgG response before 90 days. For this last group of patients, it is unknown whether the IgG response will be sustained or not. However, we also classified them as SR, building a subgroup of patients enriched for short response. Figure 4 illustrates the study subgroups and their respective sources. Hence, the LR and SR subgroups of both studies were merged, generating two final major groups, the LR group with 84 patients and the SR group with 65 patients.

The plasmatic cytokine profiles were previously described by Queiroz et al. [21] for a larger sample, but that includes the same samples of the previous IgG studies. Hence, we reappraise the cytokine data but focus on the anti-SARS-CoV-2 IgG response duration, looking for cytokine signatures that help us obtain an understanding of the IgG response dynamics. There are cytokine data on the plasmatic levels of IFN-γ, TNF-α, IL-17A, IL-6, IL-2, IL-10, and IL-4 for 64 LR and 47 SR patients.

In addition, the samples from all studies were examined for the presence of ten genetic markers that had been previously published, with a particular focus on the genetic predisposition to long COVID: *IFNG* +874T/A rs2430561; *TNF* -308G/A (rs1800629); *IL6* -174G/C (rs1800795); *IL6R* 358A/C (rs2228145); *MTHFR* C677T, Ala222Val (rs1801133); *FV Leiden* R506QC/T (rs6025); *CD209* -336A/G (rs4804803); *CIITA* -168A/G (rs3087456); *HLA-DPA1* (rs3077); and *HLA-DPB1* (rs9277534) [22]. The genotypic data were available for all 84 LR and 65 SR patients. Hence, we were able to reappraise all of these data, addressing the role of these markers in IgG response duration.

Additionally, the genotypic and allele frequencies for six SNPs (rs1800795/*IL6*; rs2430561/*IFNG*; rs1801133/*MTHFR*; rs6025/*FV*; rs4804803/*CD209*; and rs1800629/*TNF*) in the general population of Belém (control group) were obtained from studies conducted and published before the COVID-19 pandemic, as reviewed and consolidated by Silva et al. [22].

To fill the gap in data for the four remaining SNPs, without genotypic data for a representative population of Belém before the pandemic for the control group, we recalled individuals from an old case–control study published by our group and were able to genotype 92 individuals that randomly attended to this recall for these four SNPs (rs2228145/*IL6R*; rs3087456/*CIITA*; rs3077/*HLA-DPA1*; and rs9277534/*HLA-DPB1*) [53].

Hence, both approaches, retrieving SNP allele frequencies from studies published before the pandemic, and the random recall and typing of individuals from previous pre-pandemic studies allowed us to obtain a representative profile of the allele frequencies of Belém before the advent of COVID-19.

All laboratory methods used to conduct the IgG measurement by an ELISA (Dia. Pro-DiagnosticBioprobes, Milan, Italy), cytokine profiling by flow cytometry (BD Biosciences, San Diego, CA, USA), and SNP genotyping by TaqMan™ Real-Time Assays (Thermo Fisher Scientific Baltics UAB, Vilnius, Lithuania)were already described in their respective published studies [11,21,22].

Finally, aiming to survey the putative influence of immunoglobulin production in the anti-SARS-CoV-2 IgG response, the total plasmatic IgG, IgA, and IgM levels were evaluated, in the same samples, using the ELISA method, the commercial kits Human IgG Total ELISA Kit (Invitrogen™, Thermo Fisher Scientific, catalog BMS2091, Vienna, Austria), Human IgA ELISA Kit (Invitrogen™, Thermo Fisher Scientific, catalog BMS2096, Vienna, Austria), and Human IgM ELISA Kit (Invitrogen™, Thermo Fisher Scientific, catalog BMS2098, Vienna, Austria), all carried out according to the manufacturer’s instructions.

The present study was approved and registered at the National Ethic Committee (CAEE: 33470020.1001.0018; protocol number nº 2.190.330). Written informed consent forms were signed by all of the participants. This study was conducted following the principles of the Declaration of Helsinki and followed the guidelines recommended by the Strengthening the Reporting of Genetic Association Studies (STREGA) [54].

### 4.2. Statistical Analysis

Two subgroups were built with IgG long response (LR; 84 individuals) and short response (SR; 65 individuals) patients. Categorical variables were compared using Fisher’s exact test. Mann–Whitney non-parametrical test was used to compare continuous variables between both subgroups. In the case of COVID-19 symptom frequency, we used the Wilcoxon non-parametric paired test, and for correlation between IL-17A levels and the levels of IL-6, IFN-γ, IL4, and IL-2, Spearman’s non-parametric correlation was conducted. Correction for multiple tests was applied when necessary.

The IgG, IgA, and IgM values between the LR and SR subgroups were compared using the Mann–Whitney test. Additionallythe plasmatic levels of the total IgG, IgA, and IgM levels were correlated, and the anti-SARS-CoV-2 IgG values were determined using Spearman’s correlation. Each of these processes was performed separately for the Oliveira et al. and Soares et al. datasets because different commercial kits were used in each study. The graphics were generated using GraphPad Prism 9.0 (GraphPad Software, Boston, MA, USA) and Inkscape v.1.3.2 software.

## Figures and Tables

**Table 1:** Demographic, epidemiological, and clinical characterizations of long and short IgG response duration against spike protein epitopes of SARS-CoV-2.

Variables	Long Response (%)	Short Response (%)	Statistical Analysis
Sex			
Female	49 (58.3)	26 (40)	Fisher’s Exact Test; *p* = 0.03
Male	35 (41.7)	39 (60)	
Age (years)			
24–39	39 (46.4)	30 (46.1)	Mann–Whitney Test; *p* = 0.56
40–59	38 (45.2)	27 (41.5)	
≥60	7 (8.4)	8 (12.3)	
Mean	41.73	43.49	
Ethnicity			
Brown	41 (57.7)	30 (52.6)	Fisher’s Exact Test; *p* > 0.05
White	20 (28.2)	18 (31.6)	
Black	7 (9.9)	8 (14.0)	
Asian	3 (4.2)	1 (1.8)	
Not Informed	13	8	
Comorbidities			
Yes	27 (32.1)	20 (30.8)	Fisher’s Exact Test; *p* > 0.05
No	57 (67.9)	45 (69.2)	
Diabetes	9 (10.7)	9 (13.8)	
Cardiovascular	19 (22.6)	11 (17)	
Obesity	10 (12)	7 (10.7)	
Respiratory	1 (1.2)	0	
Renal	1 (1.2)	0	
Hospitalization			
No	59 (70.3)	29 (44.6)	Fisher’s Exact Test; *p* = 0.0024; *p_c_* = 0.014
Yes	25 (29.7)	36 (55.4)	
Long COVID			
No	46 (62.2)	25 (54.3)	Fisher’s Exact Test; *p* = 0.44
Yes	28 (37.8)	21 (45.7)	
Disease Severity			
Asymptomatic	2 (2.4)	1 (1.5)	Fisher’s Exact Test showed statistical differences between response groups (asymptomatic + mild versus moderate + severe; *p* = 0.0025; *p_c_* = 0.015)
Mild	57 (67.8)	28 (43.0)	
Moderate	9 (10.7)	18 (27.7)	
Severe	16 (19.0)	18 (27.7)	
Symptoms			
Fever	58 (69.0)	48 (74.0)	Wilcoxon paired test comparing proportions across all symptoms between response groups; Z = 2.53; *p* = 0.011
Cough	43 (51.2)	46 (70.8)	
Coryza	33 (39.3)	25 (38.5)	
Retroocular pain	27 (32.1)	9 (13.8)	
Headache	59 (70.3)	39 (60.0)	
Sore throat	49 (58.3)	25 (38.5)	
Chest pain	43 (51.2)	24 (37.0)	
Abdominal pain	26 (31.0)	14 (21.5)	
Myalgia	60 (71.4)	36 (55.4)	
Nausea	18 (21.4)	16 (24.6)	
Vomit	10 (12.0)	8 (12.3)	
Diarrhea	39 (46.4)	25 (38.5)	
Dyspnea	44 (52.4)	31 (47.7)	
Weakness	43 (51.2)	22 (34.0)	
Fatigue	55 (65.5)	28 (43.0)	
Anosmia	59 (70.3)	33 (50.8)	
Ageusia	61 (72.6)	32 (49.2)	

*p_c_* = *p* corrected for multiple tests.

**Table 2:** Genotype and allele frequencies for SNPs among long (LR) and short (SR) response subgroups and control subgroup.

Locus	Genotype and Alleles	Control (%)	LR (%)	SR (%)	Statistical Analysis (Fisher’s Exact Test)
*IL6*	GG	207 (69)	47 (56)	38 (58)	The comparison of the GG genotype frequencies between the control and LR was statistically significant (*p* = 0.03). The remaining comparisons between the control vs. SR and LR vs. SR did not achieve statistical significance.
	GC	85 (28)	31 (37)	25 (38)	
	CC	8 (3)	6 (7)	2 (3)	
	G	83%	74.4%	77.7%	
*IL6R*	AA	20 (22)	25 (29.7)	28 (43)	The comparison of AA genotype frequencies between the control and SR was statistically significant (*p* = 0.005). The remaining comparisons between the control vs. LR and LR vs. SR did not achieve statistical significance.
	CA	55 (60)	36 (43)	27 (41.5)	
	CC	17 (18)	23 (27.4)	10 (15.4)	
	C	48%	48.8%	36%	
*IFNG*	AA	226 (57)	37 (44)	28 (43)	The comparisons of AA genotype frequencies between the control and LR and the control and SR were statistically significant (*p* = 0.04 and *p* = 0.04, respectively). The remaining comparison between LR and SR did not achieve statistical significance.
	AT	147 (37)	34 (40)	26 (40)	
	TT	25 (6)	13 (15.5)	11 (17)	
	T	25%	35.7%	37%	
*CD209*	AA	50 (69)	57 (68)	40 (61.5)	*p* > 0.05
	AG	19 (26)	26 (31)	24 (37)	
	GG	3 (4)	1 (1.2)	1 (1.5)	
	G	17%	16.6%	20%	
*TNF*	GG	390 (78)	73 (87)	54 (83)	*p* > 0.05
	AG	96 (19)	11 (13)	11 (17)	
	AA	11 (2)	0 (0)	0 (0)	
	A	12%	6.5%	8.4%	
*DPA1*	AA	40 (44)	43 (51.2)	28 (43.1)	*p* > 0.05
	AG	38 (41.7)	32 (38.1)	31 (47.7)	
	GG	13 (14.3)	9 (10.7)	6 (9.2)	
	A	64%	70%	67%	
*DPB1*	AA	34 (37)	37 (44)	25 (38)	*p* > 0.05
	AG	41 (45)	36 (43)	33 (51)	
	GG	17 (18)	11 (13)	7 (11)	
	A	59%	65.5%	64%	
*CIITA*	AA	29 (32)	20 (24)	17 (26)	*p* > 0.05
	AG	43 (47)	41 (49)	26 (40)	
	GG	19 (21)	23 (27)	22 (34)	
	A	55%	48.2%	46%	
*FV*	CC	123 (97)	81 (96.4)	65 (100)	*p* > 0.05
	CT	4 (3)	3 (3.6)	0 (0)	
	TT	0 (0)	0	0 (0)	
	T	1.6%	1.8%	0%	
*MTHFR*	GG	56 (44)	33 (39)	27 (41)	*p* > 0.05
	AG	56 (44)	42 (50)	29 (45)	
	AA	15 (12)	9 (11)	9 (14)	
	A	34%	36%	36%	

**Table 3:** The correlation between the IgG anti-SARS-CoV-2 and plasmatic levels of total immunoglobulin G, A, and M (IgG, IgA, and IgM) among patients. The correlation was carried out separately with data from Oliveira et al.’s and Soares et al.’s data sources. Spearman’s correlation coefficient (rs) and the respective *p*-values are presented. No statistical significance was achieved in any correlation tested.

Immunoglobulin Plasmatic Levels	Oliveira et al. [10]	Soares et al. [9]
Total IgG	rs = −0.05; *p* = 0.70	rs = −0.13; *p* = 0.19
Total IgA	rs = −0.20; *p* = 0.12	rs = −0.0026; *p* = 0.98
Total IgM	rs = −0.01; *p* = 0.94	rs = 0.13; *p* = 0.19

## Data Availability

The raw data supporting the conclusions of this article will be made available by the authors without undue reservation.

## Supplementary Materials

The following supporting information can be downloaded at: https://www.mdpi.com/article/10.3390/ijms25168740/s1.
