# Supplementary material for: Genetic, Clinical, Epidemiological, and Immunological Profiling of IgG Response Duration after SARS-CoV-2 Infection
Source: Int J Mol Sci. 2024 Aug 10;25(16):8740. doi: 10.3390/ijms25168740 (PMC11354850; doi:10.3390/ijms25168740)
Supplement: Supplementary file 1 [file ijms-25-08740-s001.zip › ijms-3066330-supplementary.pdf]

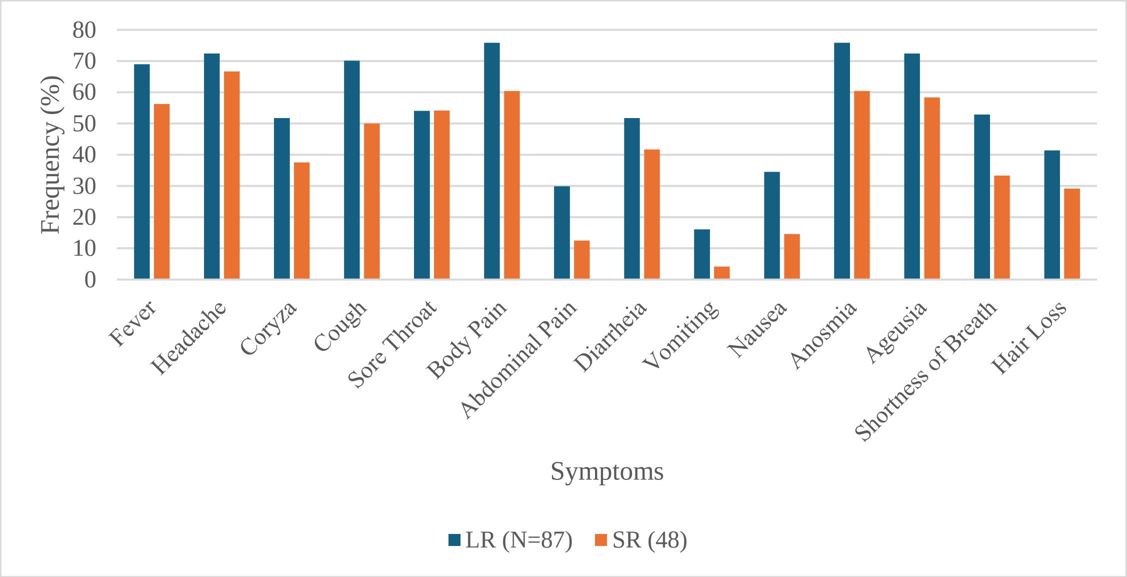

Figure S1. Frequency (%) of symptoms reported by Bichara et al. [8]. LR (Long Response) frequencies were obtained from the Bichara´s paper Table 5 (Persistent) and the SR (Short Response) were obtained from the sum of Table 5's Nonpersistent plus the Nonreactive from Bichara´s paper Table 2. (Wilcoxon paired test; Z=3.23; p=0.0012).

Table S1. COVID-19 Symptoms in Belém, Pará, Brazil population.

<table>
<tr><th>Severity</th><th>Nonsevere</th><th>Severe</th><th>Non-Hospitalized</th><th>Hospitalized</th></tr>
<tr><td>Sample Sizes</td><td>N</td><td>N</td><td>N</td><td>N</td></tr>
<tr><td>References</td><td colspan="2">Sarges et al.[18]</td><td colspan="2">Rodrigues et al.[19]</td></tr>
<tr><td>Symptoms</td><td></td><td></td><td></td><td></td></tr>
<tr><td>Fever</td><td>69.33</td><td>73.61</td><td>61.05</td><td>75</td></tr>
<tr><td>Cough</td><td>62</td><td>84.72</td><td>48.42</td><td>81.81</td></tr>
<tr><td>Runny nose/Coryza</td><td>40.67</td><td>33.33</td><td>40</td><td>35.61</td></tr>
<tr><td>Headache</td><td>58</td><td>58.33</td><td>61.05</td><td>53.79</td></tr>
<tr><td>Sore throat</td><td>40</td><td>31.94</td><td>42.1</td><td>32.57</td></tr>
<tr><td>Chest pain</td><td>40</td><td>52.78</td><td>31.58</td><td>42.42</td></tr>
<tr><td>Abdominal pain</td><td>16.67</td><td>30.56</td><td>16.84</td><td>23.48</td></tr>
<tr><td>Muscle or body pain</td><td>58.67</td><td>58.33</td><td>53.68</td><td>59.85</td></tr>
<tr><td>Nausea</td><td>19.33</td><td>37.5</td><td rowspan="2">17.89</td><td rowspan="2">34.09</td></tr>
<tr><td>Vomiting</td><td>10</td><td>15.28</td></tr>
<tr><td>Diarrhea</td><td>42.67</td><td>44.44</td><td>34.74</td><td>47.73</td></tr>
<tr><td>Dyspnea</td><td>36.67</td><td>73.61</td><td>29.47</td><td>60.61</td></tr>
<tr><td>Weakness</td><td>49.33</td><td>65.28</td><td rowspan="2">20</td><td rowspan="2">27.27</td></tr>
<tr><td>Fatigue</td><td>54.67</td><td>75</td></tr>
<tr><td>Anosmia</td><td>52.67</td><td>38.89</td><td rowspan="2">63.16</td><td rowspan="2">39.39</td></tr>
<tr><td>Ageusia</td><td>50.67</td><td>38.89</td></tr>
</table>
